# Supplementary material for: Utilization of Primary Healthcare Services in Patients with Multimorbidity According to Their Risk Level by Adjusted Morbidity Groups: A Cross-Sectional Study in Chamartín District (Madrid)
Source: Healthcare (Basel). 2024 Jan 20;12(2):270. doi: 10.3390/healthcare12020270 (PMC10815081; doi:10.3390/healthcare12020270)
Supplement: Supplementary file 1 [file healthcare-12-00270-s001.zip › healthcare-2751757-supplementary.pdf]

SUPPLEMENTARY MATERIAL

Table S1. Types of chronic diseases considered by the Adjusted Morbidity Group (AMG) in the Community of Madrid at the time of data extraction.

Alcohol abuse	Hypertension
Anaemia	Ischemic heart disease
Aorta aneurysm	Leukemia
Anxiety	Liver cancer
Arthritis	Lung cancer
Arthrosis	Mental retardation
Asthma	Multiple sclerosis
Attention-Deficit/Hyperactivity Disorder	Obesity
Bladder cancer	Obstructive chronic pulmonary disease (OCPD)
Breast cancer	Osteoarthritis
Cardiopulmonary disease	Osteoporosis
Central nervous system cancer	Pancreatic cancer
Cervical cancer	Parkinson
Cirrhosis	Prostate cancer
Colon cancer	Renal cancer
Dementia	Renal chronic failure
Depression	Retinoblastoma
Diabetes Mellitus	Schizophrenia
Dyslipidemia	Skin cancer
Dysrhythmias	Soft tissues cancer
Ear, nose and throat cancer	Stomach cancer
Endometrial cancer	Stroke
Epilepsy	Substance abuse
Gastrointestinal ulcer	Testicle cancer
Glaucoma	Thyroid cancer
Heart chronic failure	Thyroid disorder
Hepatoblastoma	Ulcerative colitis
Hodgkin/Other lymphomas	Valvular heart disease
Human immunodeficiency virus (HIV)	Vasculitis
Hyperlipidemia	

Table S2. Sociodemographic, functional and clinical characteristics of the patients with multimorbidity by risk level according to Adjusted Morbidity Groups.

Multimorbidity patients n (%)	High risk 440 (7.3)	Medium risk 1,721 (28.5)	Low risk 3,875 (64,2)	p-value
**Sociodemographic variables**				
Female	231 (52.5)	1120 (65.1)	2480 (64.0)	<0.001
Age*	78.2 (12.4)	72.8 (14.1)	58.6 (17.1)	<0.001
≤ 65 years	67 (15.2)	436 (25.3)	2472 (63.8)	<0.001
> 65 years	373 (84.8)	1285 (74.7)	1403 (36.2)	
Origin Spain	384 (87.3)	1519 (88.3)	3149 (81.3)	<0.001
Europe	8 (1.8)	45 (2.6)	136 (3.5)	
Rest of the world	48 (10.9)	157 (9.1)	590 (15.2)	
**Functional variables**				
Immobilized	125 (28.4)	124 (7.2)	39 (1.0)	<0.001
Institutionalized	42 (9.5)	52 (3.0)	51 (1.3)	<0.001
Primary caregiver	101 (23.0)	99 (5.8)	20 (0.5)	<0.001
Home support	29 (6.6)	38 (2.2)	10 (0.3)	<0.001
Palliative care	29 (6.6)	6 (0.3)	6 (0.2)	<0.001
**Clinical variables**				
Complexity weight*	30.3 (11.9)	12.5 (2.7)	5.2 (2.0)	<0.001
Chronic diseases*	6.8 (2.3)	4.4 (1.5)	2.7 (0.9)	<0.001
Polymedicated	351 (79.8)	759 (44.1)	418 (10.8)	<0.001

* Mean (standard deviation).

Table S3. Sociodemographic, functional and clinical characteristics of the patients with multimorbidity by sex and age group.

Multimorbidity patients	Female	Male	p-value	Age ≤ 65	Age > 65	p-value
**n (%)**	**3,831 (63.5)**	**2,205 (36,5)**		**2,975 (49.3)**	**3,061 (50.7)**	
**Sociodemographic variables**						
Female	-	-	-	1,779 (59.8)	2,052 (67.0)	<0.001
Age*	65.2 (17.7)	62.1 (17.3)		49.4 (11.8)	78.3 (8.1)	<0.001
≤ 65 years	1,779 (46.4)	1,196 (54.2)	<0.001	-	-	-
> 65 years	2,052 (53.6)	1,009 (45.8)				
Origin Spain	3,198 (83.5)	1854 (84.1)		2447 (82.3)	2,605 (85.1)	
Europe	125 (3.3)	64 (2.9)	0.703	103 (3.5)	86 (2.8)	0.011
Rest of the world	508 (13.3)	287 (13.0)		425 (14.3)	370 (12.1)	
**Functional variables**						
Immobilized	213 (5.6)	75 (3.4)	<0.001	9 (0.3)	279 (9.1)	<0.001
Institutionalized	108 (2.8)	37 (1.7)	0.005	3 (0.1)	142 (4.6)	<0.001
Primary caregiver	156 (4.1)	64 (2.9)	0.020	8 (0.3)	212 (6.9)	<0.001
Home support	55 (1.4)	22 (1.0)	0.144	1 (0.0)	76 (2.5)	<0.001
Palliative care	19 (0.5)	22 (1.0)	0.022	9 (0.3)	32 (1.0)	<0.001
**Clinical variables**						
High Risk Level	231 (6.0)	209 (9.5)		67 (2.3)	373 (12.2)	
Medium	1,120 (29.2)	601 (27.3)	<0.001	436 (14.7)	1,285 (42.0)	<0.001
Low	2,480 (64.7)	1,395 (63.3)		2,472 (83.1)	1,403 (45.8)	
Complexity weight*	8.8 (7.2)	9.6 (8.7)	<0.001	6.4 (5.1)	11.8 (8.9)	<0.001
Chronic diseases*	3.6 (1.7)	3.4 (1.7)	0,001	2.8 (1.2)	4.1 (1.9)	<0.001
Polymedicated	1,059 (27.6)	469 (21.3)	<0.001	32 (1.1)	1496 (48.9)	<0.001

* Mean (standard deviation).

Table S4. Comorbidities of the patients with multimorbidity by risk level according to Adjusted Morbidity Groups.

**Multimorbidity patients**		**High risk**	**Medium risk**	**Low risk**	**p-value**
	**n (%)**	**440 (7.3)**	**1,721 (28.5)**	**3,875 (64,2)**	
Haematic comorbidity	Anemia	118 (26.8)	164 (9.5)	336 (8.7)	<0.001
	HIV	6 (1.4)	11 (0.6)	19 (0.5)	0.076
Digestive comorbidity	Cirrhosis	50 (11.4)	187 (10.9)	197 (5.1)	<0.001
	Inflammatory bowel disease	4 (0.9)	20 (1.2)	27 (0.7)	0.212
	Gastric ulcer	23 (5.2)	47 (2.7)	84 (2.2)	0.001
	Chronic pancreatitis	2 (0.5)	2 (0.1)	3 (0.1)	0.088
	Cystic fibrosis	2 (0.5)	0 (0.0)	1 (0.0)	<0.001
Ocular comorbidity	Glaucoma	45 (10.2)	154 (8.9)	157 (4.1)	<0.001
Cardiovascular comorbidity	Hypertension	364 (82.7)	1187 (69.0)	1582 (40.8)	<0.001
	Dysrhythmias	192 (43.6)	298 (17.3)	160 (4.1)	<0.001
	Chronic heart failure	123 (28.0)	101 (5.9)	14 (0.4)	<0.001
	Coronary disease	111 (25.2)	171 (9.9)	77 (2.0)	<0.001
	Valvular heart disease	80 (18.2)	66 (3.8)	39 (1.0)	<0.001
Musculoskeletal comorbidity	Osteoarthritis	108 (24.5)	425 (24.7)	430 (11.1)	<0.001
	Osteoporosis	103 (23.4)	423 (24.6)	508 (13.1)	<0.001
	Arthritis	24 (5.5)	94 (5.5)	70 (1.8)	<0.001
	Lupus	3 (0.7)	0 (0.0)	1 (0.0)	0.002
	Vasculitis	6 (1.4)	7 (0.4)	13 (0.3)	0.008
Neurological comorbidity	Dementia	56 (12.7)	91 (5.3)	53 (1.4)	<0.001
	Stroke	92 (20.9)	112 (6.5)	48 (1.2)	<0.001
	Parkinson	16 (3.6)	46 (2.7)	22 (0.6)	<0.001
	Epilepsy	22 (5.0)	41 (2.4)	64 (1.7)	<0.001
	Multiple sclerosis	0 (0)	5 (0.3)	13 (0.3)	<0.001
Psychiatric comorbidity	Alcohol abuse	198 (5.1)	110 (6.4)	44 (10.0)	<0.001
	Substance abuse	6 (1.4)	28 (1.6)	66 (1.7)	0.864
	Anxiety	84 (19.1)	417 (24.2)	1166 (30.1)	<0.001
	Depression	101 (23.0)	386 (22.4)	583 (15.0)	<0.001
	Bipolar illness	4 (0.9)	17 (1.0)	35 (0.9)	0.954
	Psychotic disorder	5 (1.1)	24 (1.4)	36 (0.9)	0.295
Respiratory comorbidity	COPD	109 (24.8)	163 (9.5)	95 (2.5)	<0.001
	Asthma	26 (5.9)	167 (9.7)	407 (10.5)	0.009
Endocrine comorbidity	Dyslipidemia	298 (67.7)	1072 (62.3)	1919 (49.5)	<0.001
	Diabetes mellitus	189 (43.0)	432 (25.1)	382 (9.9)	<0.001
	Obesity	131 (29.8)	463 (26.9)	791 (20.4)	<0.001
	Thyroid disorder	107 (24.3)	406 (23.6)	823 (21.2)	0.077
Renal comorbidity	Renal chronic failure	97 (22.0)	36 (2.1)	7 (0.2)	<0.001
	Repeat urinary tract infection	85 (19.3)	139 (8.1)	123 (3.2)	<0.001
Cancer comorbidity	Any cancer	164 (37.3)	176 (10.2)	87 (2.2)	<0.001
	Breast	27 (6.1)	23 (1.3)	12 (0.3)	<0.001
	Prostate	24 (5.5)	31 (1.8)	6 (0.2)	<0.001
	Skin	13 (3.0)	20 (1.2)	19 (0.5)	<0.001
	Colorectal	20 (4.5)	27 (1.6)	6 (0.2)	<0.001
	Bladder	16 (3.6)	13 (0.8)	5 (0.1)	<0.001
	Lung	20 (4.5)	11 (0.6)	2 (0.1)	<0.001
	Cervix	3 (0.7)	3 (0.2)	9 (0.2)	0.153
	Liver	2 (0.5)	3 (0.2)	0 (0.0)	0.002
	Gastric	5 (1.1)	3 (0.2)	0 (0.0)	<0.001
	Pancreas	4 (0.9)	2 (0.1)	0 (0.0)	<0.001
	Renal	7 (1.6)	3 (0.2)	2 (0.1)	<0.001
	Endometrium	2 (0.5)	1 (0.1)	2 (0.1)	0.019
	Leukaemia	9 (2.0)	14 (0.8)	2 (0.1)	<0.001
	Lymphoma	15 (3.4)	19 (1.1)	13 (0.3)	<0.001

COPD: Chronic obstructive pulmonary disease.

Table S5. Comorbidities of the patients with multimorbidity by sex and age group.

Multimorbidity patients		Female	Male	p-value	Age ≤ 65	Age > 65	p-value
n (%)		3,831 (63.5)	2,205 (36,5)		2,975 (49.3)	3,061 (50.7)	
Haematic comorbidity	Anemia	469 (12.2)	149 (6.8)	<0.001	351 (11.8)	267 (8.7)	<0.001
	HIV	6 (0.2)	30 (1.4)	<0.001	32 (1.1)	4 (0.1)	<0.001
Digestive comorbidity	Cirrhosis	230 (6.0)	204 (9.3)	<0.001	223 (7.5)	211 (6.9)	0.365
	Inflammatory bowel disease	26 (0.7)	25 (1.1)	0.063	25 (0.8)	26 (0.8)	0.969
	Gastric ulcer	71 (1.9)	83 (3.8)	<0.001	64 (2.2)	90 (2.9)	0.052
	Chronic pancreatitis	4 (0.1)	3 (0.1)	0.728	3 (0.1)	4 (0.1)	0.733
	Cystic fibrosis	1 (0.0)	2 (0.1)	<0.001	3 (0.1)	0 (0.0)	0.079
Ocular comorbidity	Glaucoma	239 (6.2)	117 (5.3)	<0.001	76 (2.6)	280 (9.1)	<0.001
Cardiovascular comorbidity	Hypertension	1872 (48.9)	1258 (57.1)	<0.001	926 (31.1)	2207 (72.1)	<0.001
	Dysrhythmias	388 (10.1)	262 (11.9)	0.034	131 (4.4)	519 (17.0)	<0.001
	Chronic heart failure	150 (3.9)	88 (4.0)	0.885	18 (0.6)	220 (7.2)	<0.001
	Coronary disease	136 (3.5)	223 (10.1)	<0.001	71 (2.4)	288 (9.4)	<0.001
	Valvular heart disease	113 (2.9)	72 (3.3)	0.493	36 (1.2)	149 (4.9)	<0.001
Musculoskeletal comorbidity	Osteoarthritis	753 (19.7)	210 (9.5)	<0.001	262 (8.8)	701 (22.9)	<0.001
	Osteoporosis	990 (25.8)	44 (2.0)	<0.001	232 (7.8)	802 (26.2)	<0.001
	Arthritis	129 (3.4)	59 (2.7)	<0.001	91 (3.1)	97 (3.2)	0.806
	Lupus	2 (0.1)	2 (0.1)	0.576	1 (0.0)	3 (0.1)	0.331
	Vasculitis	21 (0.5)	5 (0.2)	0.066	8 (0.3)	18 (0.6)	0.058
Neurological comorbidity	Dementia	152 (4.0)	48 (2.2)	<0.001	7 (0.2)	193 (6.3)	<0.001
	Stroke	139 (3.6)	113 (5.1)	0.005	44 (1.5)	208 (6.8)	<0.001
	Parkinson	44 (1.1)	40 (1.8)	0.034	7 (0.2)	77 (2.5)	<0.001
	Epilepsy	70 (1.8)	57 (2.6)	0.048	71 (2.4)	56 (1.8)	0.132
	Multiple sclerosis	15 (0.4)	3 (0.1)	0.080	15 (0.5)	3 (0.1)	0.004
Psychiatric comorbidity	Alcohol abuse	93 (2.4)	259 (11.7)	<0.001	242 (8.1)	110 (3.6)	<0.001
	Substance abuse	30 (0.8)	70 (3.2)	<0.001	95 (3.2)	5 (0.2)	<0.001
	Anxiety	1211 (31.6)	456 (20.7)	<0.001	1065 (35.8)	602 (19.7)	<0.001
	Depression	821 (21.4)	249 (11.3)	<0.001	545 (18.3)	525 (17.2)	0.235
	Bipolar illness	34 (0.9)	22 (1.0)	0.667	42 (1.4)	14 (0.5)	<0.001
	Psychotic disorder	35 (0.9)	30 (1.4)	0.105	49 (1.6)	16 (0.5)	<0.001
Respiratory comorbidity	COPD	160 (4.2)	207 (9.4)	<0.001	101 (3.4)	266 (8.7)	<0.001
	Asthma	425 (11.1)	175 (7.9)	<0.001	426 (14.3)	174 (5.7)	<0.001
Endocrine comorbidity	Dyslipidemia	1988 (51.9)	1301 (59.0)	<0.001	1350 (45.6)	1939 (63.3)	<0.001
	Diabetes mellitus	520 (13.6)	483 (21.9)	<0.001	287 (9.6)	716 (23.4)	<0.001
	Obesity	833 (21.7)	552 (25.0)	0.003	689 (23.2)	696 (22.7)	0.697
	Thyroid disorder	1116 (29.1)	220 (10.0)	<0.001	675 (22.7)	661 (21.6)	0.306
Renal comorbidity	Renal chronic failure	70 (1.8)	70 (3.2)	0.001	12 (0.4)	128 (4.2)	0.331
	Repeat urinary tract infection	278 (7.3)	69 (3.1)	<0.001	131 (4.4)	216 (7.1)	<0.001
Cancer comorbidity	Any cancer	216 (5.6)	211 (9.6)	<0.001	135 (4.5)	292 (9.5)	<0.001
	Breast	61 (1.6)	1 (0.0)	<0.001	25 (0.8)	37 (1.2)	0.156
	Prostate	0 (0.0)	61 (2.8)	<0.001	9 (0.3)	52 (1.7)	<0.001
	Skin	32 (0.8)	20 (0.9)	0.772	15 (0.5)	37 (1.2)	0.003
	Colorectal	24 (0.6)	29 (1.3)	0.006	14 (0.5)	39 (1.3)	0.001
	Bladder	5 (0.1)	29 (1.3)	<0.001	10 (0.3)	24 (0.8)	0.020
	Lung	13 (0.3)	20 (0.9)	0.004	9 (0.3)	24 (0.8)	0.011
	Cervix	15 (0.4)	0 (0.0)	0.003	13 (0.4)	2 (0.1)	0.004
	Liver	2 (0.1)	3 (0.1)	0.276	0 (0.0)	5 (0.2)	0.027
	Gastric	3 (0.1)	5 (0.2)	0.127	1 (0.0)	7 (0.2)	0.037
	Pancreas	2 (0.1)	4 (0.2)	0.125	1 (0.0)	5 (0.2)	0.110
	Renal	3 (0.1)	9 (0.4)	0.006	3 (0.1)	9 (0.3)	0.092
	Endometrium	5 (0.1)	0 (0.0)	0.90	3 (0.1)	2 (0.1)	0.632
	Leukaemia	11 (0.3)	14 (0.6)	0.043	6 (0.2)	19 (0.6)	0.011
	Lymphoma	31 (0.8)	16 (0.7)	0.722	13 (0.4)	34 (1.1)	0.003

COPD: Chronic obstructive pulmonary disease.

Table S6. Annual primary care services utilization in patients with multimorbidity by risk level according to Adjusted Morbidity Groups.

**Primary care contacts**	**High risk**	**Medium risk**	**Low risk**	**p-value**
**Mean (SD)**	**440 (7.3)**	**1,721 (28.5)**	**3,875 (64,2)**	
Total Annual Contacts	34.1 (28.0)	21.5 (17.4)	9.8 (10.4)	<0.001
Type of contact				
Health related	29.1 (25.8)	18.5 (15.1)	8.4 (8.9)	<0.001
Administrative	3.3 (6.8)	1.8 (4.8)	0.7 (2.6)	<0.001
Laboratory	1.8 (2.4)	1.2 (1.5)	0.7 (1.1)	<0.001
Form of contact				
Face-to-face	26.7 (20.7)	19.2 (15.0)	9.4 (9.9)	<0.001
Telephone	2.4 (7.9)	0.9 (2.8)	0.2 (0.7)	<0.001
Home visit	5.3 (13.0)	1.5 (5.1)	0.2 (1.9)	<0.001
Professional contacted				
Doctor	15.9 (11.9)	11.0 (8.3)	5.4 (5.2)	<0.001
Nurse	12.9 (18.6)	6.6 (8.5)	2.5 (4.5)	<0.001
Physiotherapist	0.00 (0.00)	0.6 (2.8)	0.3 (2.0)	0.002
Midwife	0.01 (0.1)	0.04 (0.4)	0.1 (0.5)	0.004
Dentist	0.00 (0.00)	0.06 (0.5)	0.04 (0.3)	0.120
Social worker	0.3 (1.5)	0.2 (1.0)	0.04 (0.4)	<0.001

SD: Standard Deviation.

Table S7. Annual primary care services utilization in patients with multimorbidity by sex and age group.

Primary care contacts Mean (SD)	Female 3,831 (63.5)	Male 2,205 (36,5)	p-value	Age ≤ 65 2,975 (49.3)	Age > 65 3,061 (50.7)	p-value
Total Annual Contacts	15.3 (15.7)	14.3 (17.6)	0.011	10.1 (12.3)	19.6 (18.4)	<0.001
Type of contact						
Health related	13.2 (13.6)	12.1 (15.5)	0.041	8.5(10.3)	16.9 (16.3)	<0.001
Administrative	1.1 (3.7)	1.3 (4.2)	<0.001	0.8 (2.9)	1.6 (4.6)	<0.001
Laboratory	1.0 (1.5)	0.8 (1.3)	0.006	0.7 (1.1)	1.1 (1.6)	<0.001
Form of contact						
Face-to-face	13.7 (13.2)	13.0 (14.8)	0.056	9.7 (11.6)	17.1 (14.8)	<0.001
Telephone	0.6 (3.0)	0.5 (2.2)	0.128	0,2 (1.5)	0.8 (3.5)	<0.001
Home visit	1.0 (4.2)	0.9 (5.8)	0.015	0.1 (1.2)	1.7 (6.6)	<0.001
Professional contacted						
Doctor	8.2 (7.9)	7.1 (7.2)	<0.001	5.8 (6.4)	9.7 (8.3)	<0.001
Nurse	4.3 (7.0)	4.6 (10.0)	0.021	2.2 (5.4)	6.5 (9.8)	<0.001
Physiotherapist	0.4 (2.4)	0.3 (1.8)	0.398	0.3 (1.9)	0.4 (2.4)	0.094
Midwife	0.1 (0.5)	0.00 (0.02)	<0.001	0.1 (0.6)	0.01 (0.1)	<0.001
Dentist	0.04 (0.4)	0.05 (0.4)	0.656	0.06 (0.5)	0.03 (0.3)	<0.001
Social worker	0.1 (0.8)	0.1 (0.7)	0.015	0.03 (0.3)	0.2 (1.0)	<0.001

SD: Standard Deviation.
